# Association of rs8670 Polymorphism in the MSX1 Gene With Non-Syndromic Cleft Lip With or Without Cleft Palate in Malay Population

**DOI:** 10.7759/cureus.68958

**Published:** 2024-09-08

**Authors:** Roslina Rashid, Zainul Ahmad Rajion, Bin Alwi Zilfalil, Saidi Jaafar

**Affiliations:** 1 Basic Sciences Unit, School of Dental Sciences, Universiti Sains Malaysia, Kubang Kerian, MYS; 2 Kulliyyah of Dentistry, International Islamic University Malaysia, Kuantan, MYS; 3 Human Genome Center, School of Medical Sciences, Universiti Sains Malaysia, Kubang Kerian, MYS

**Keywords:** case control studies, cleft lip with or without cleft palate, genetic polymorphisms, genetic variant, msx1

## Abstract

Objective: This study aimed to investigate the association between variants present in the MSX1 gene and the risk of developing non-syndromic cleft lip with or without cleft palate (NSCL±P) among individuals of Malay ethnicity in Malaysia.

Materials and methods: This case-control study involved 89 patients with NSCL±P and 100 healthy control subjects. Polymerase chain reaction (PCR) was performed on both exon 1 and exon 2 of the MSX1 gene using four pairs of primers. The amplification products were then subjected to denaturing high-pressure liquid chromatography for initial screening, and the presence of a heteroduplex peak was validated using direct sequencing analysis to detect the single-nucleotide polymorphism.

Results: Five previously known variations (*c.-36G>A*, *p*.*Ala30Ala*, *p.Ala34Gly*, *p*.*Gly110Gly,* and *rs8670: C>T*) were detected within the MSX1 gene in both NSCL±P patients and controls.A significant association was found between the *rs8670: C>T* variant and NSCL±P (*p *= 0.017; OR: 0.368; 95% CI: 0.152 - 0.893), with this particular single-nucleotide polymorphism present in 20% (20) among controls and 7.9% (7) of the NSCL±P cases.

Conclusions: Our data showed a lower incidence of the *rs8670: C>T* polymorphism among NSCL±P cases compared to control in this Malay population. However, since this variant is located in the 3’UTR, it could potentially impact the stability of *MSX1* mRNA.

## Introduction

Orofacial clefts are among the most common congenital birth defects, affecting about 1 in every 500 to 1 in every 1000 newborns, with considerable geographical variation in live birth prevalence [1,2]. In Malaysia, the rate of occurrence of cleft is 1 in 591 live births [3]. Cleft lip with or without cleft palate (CL±P) is a complex multifactorial disorder caused by a single or a combination of both genetic influences and environmental factors. About 70% of all CL±P cases are non-syndromic, while the remaining 30% are associated with additional characteristics, including abnormalities in other parts of the body, as seen in patients with Van der Woude syndrome, Pierre Robin syndrome, and del(22q) syndrome [4]. Classic linkage analyses with families co-segregating for prominent syndromes with cleft have been used to identify several candidate genes for CL±P, including PTCH1, MID1, PVRL1, MSX1, p63, and IRF6 [5,6].

The MSX1 gene, which encodes a protein of 297 amino acids, contains two exons separated by an intron of approximately 1.6 kb [7]. The MSX1 protein plays an important role in the development of craniofacial primordia, including tissues involved in tooth development [8,9]. Mutations in this gene have been associated with non-syndromic CL±P (NSCL±P) in various ethnicities and have been extensively studied in Asian populations [7,10-14] ]; however, the role of MSX1 variants in Malay populations has not been thoroughly examined.

The objective of this study was to further investigate the genetic contributions of the MSX1 gene to the development of non-syndromic cleft lip with or without cleft palate (NSCL±P) in the Malay population. This was achieved by screening the coding regions of the MSX1 gene using denaturing high-performance liquid chromatography (dHPLC) and direct sequencing methods to complement previously reported genetic association studies of TGFa, TGFb3, Jagged 2, and MSX1 genes within the Malay population [13,15,16].

## Materials and methods

All procedures involving human subjects were in accordance with the ethical standards of the Human Research Ethics Committee (JEPeM), Universiti Sains Malaysia (USM) [USMKK/PPSP/JK-P&E 2005] and with the Helsinki Declaration of 1975 (as revised in 2000). The selection of samples was based on inclusion and exclusion criteria set for this study, and written informed consent was obtained from all participants before their peripheral blood was collected for genetic analysis. The inclusion criteria were NSCL±P patients of any age who were of Malay ethnicity. Subjects were excluded if they met any of the following criteria: individuals with cleft palate only (CPO), as this malformation differs in genetic etiology and embryological development from CL±P, or those who had taken any epileptic medication. Based on the sample size calculation using power and samples size (PS) software version 2.131, 89 NSCL±P Malay patients were selected from the Reconstructive Science Clinic, Hospital USM, and a total of 100 healthy Malay subjects were recruited as a comparative control group. All participants were unrelated to each other and were randomly selected among patients and healthy controls.

Blood samples were collected from all participants in blood collection tubes containing EDTA. Genomic DNA was then extracted using the GeneAll Blood SV Mini kit (General Biosystem, South Korea) according to the manufacturer’s protocol. Polymerase chain reaction (PCR) was performed to amplify the two coding regions of the MSX1 gene using a previously described set of DNA primers [17]. Exon 1 of the MSX1 1.2 region and exon 2 of the MSX1 2.1 and 2.2 regions were amplified using 50 ng of genomic DNA, 0.1 µM of forward and reverse primers, 1.5 µl 10X PCR buffer II, 0.375mM dNTPs, 1.9mM MgCl2, and 1U of DNA Taq polymerase (AmpliTaq Gold®, Applied Biosystems, CA, USA) in a total volume of 20µl. Due to its high GC content, exon 1 of the MSX1 1.1 region was amplified with the addition of 10% dimethyl sulfoxide (DMSO). 

The thermocycling conditions applied were as follows: 5 minutes at 96°C for the initial denaturation step, followed by 39 cycles of denaturation at 95°C for 1 minute, annealing at 63°C for 1 minute, elongation at 72°C for 90 seconds, and a final extension at 72°C for 10 minutes. The amplified PCR products were then electrophoresed on a 1% agarose gel to confirm the presence of the correct amplicons, i.e., 421 bp for MSX1 1.1, 152 bp for MSX1 1.2, 493 bp for MSX1 2.1, and 264 bp for the MSX1 2.2 region. All amplicons, except for exon 1 of the MSX1 1.1 region, which was directly sequenced due to the use of DMSO, were screened using a Helix System 1 dHPLC (Varian Inc., Palo Alto, CA, USA) to detect unknown mutations in the exons of the MSX1 gene. dHPLC offers high sensitivity and cost-effectiveness in screening for a wide range of genetic variations. The appearance of a homoduplex peak was considered indicative of the absence of variation, while a heteroduplex peak indicated the presence of variations. Samples with aberrant dHPLC heteroduplex peaks were purified and sequenced using an ABI Prism 3100 genetic analyzer (Applied Biosystems, Foster City, CA, USA) to confirm the dHPLC results. The sequencing data were analyzed using the BioEdit program (version v7.0.9) against the MSX1 reference sequence (Accession No. AF426432) obtained from the National Centre for Biotechnology Information (NCBI).

Statistical analysis

Statistical analysis of the data was performed using IBM Corp. Released 2010. IBM SPSS Statistics for Windows, Version 19.0. Armonk, NY: IBM Corp. The statistical differences of variants between NSCL±P patients and control subjects were analyzed using Chi-square and Fisher’s exact tests, and the associated risk of the variants was estimated using a simple logistic regression test. The prevalence of variants in all regions of MSX1 was determined with a 95% confidence interval (CI) to indicate the reliability of the estimation for population parameters.

## Results

Facial clefts can be classified and divided into four distinctive groups: Group I: cleft of the lip (either unilateral or bilateral); Group II: cleft of the palate that involves only the secondary palate from posterior to the incisive foramen of the palate; Group III: cleft lip and cleft palate that occur together; Group IV: cleft palate involving the primary palate from anterior to the incisive foramen of the palate [18]. The distribution of CL±P types in this study showed that the majority of cleft patients (57.3%) were categorized under Group III, presenting with unilateral cleft lip and palate (UCLP). Of these, 34 (38.2%) had left UCLP and 17 (19.1%) had right UCLP. The remaining subjects had bilateral CLP (16; 18%), right UCL (11; 12.4%), left UCL (8; 9%), and bilateral CL (3; 3.4%) (Table 1).

**Table 1:** Distribution of cleft patients according to laterality

Clinical features of non-syndromic cleft lip with or without cleft palate (CL±P) n = 89	n (%)
Right unilateral cleft lip (R-UCL)	11 (12.3)
Left unilateral cleft lip (L-UCL)	8 (9.0)
Right unilateral cleft lip with cleft palate (R-UCLP)	17 (19.1)
Left unilateral cleft lip with cleft palate (L-UCLP)	34 (38.2)
Bilateral cleft lip (BCL)	3 (3.4)
Bilateral cleft lip with cleft palate (BCLP)	16 (18.0)

Based on dHPLC analysis, 54.8% of heteroduplex peaks were detected in the coding regions of exon 1 of the MSX1 gene for both control and patient groups. Meanwhile, only 2% of heteroduplex peaks were found in the control group in the 5’UTR, and 26.9% were detected near the 3’UTR in both patients and controls of the MSX1 DNA sequence. Another 16.3% of dHPLC results showed homoduplex peaks detected in both patients and control groups. Five previously reported common variants, including c.-36G>A in the 5’ UTR, p.Ala30Ala, p.Ala34Gly, p.Gly110Gly in exon 1, and rs8670: C>T in the 3’ UTR, were observed in both cases and controls. 

DNA sequencing analysis of MSX1 gene exon 1 detected the substitution of G to A at position 434, the substitution of C to A at position 559, a nucleotide change from C to G at position 570, and a nucleotide change from C to T at position 799. The rs8670 polymorphism, involving a nucleotide change from C to T at position 3695 in exon 2 of the MSX1 gene, was also detected.

Further analysis of genotypes and allele association in both cases and controls, conducted using simple logistic regression, revealed no significant differences for four variants: c.-36G>A, 90 C>A (p. Ala30Ala), 101C>G (p.Ala34Gly), and 330C>T (p.Gly110Gly) (Table 2). However, a significant association was found between the rs8670: C>T variant and NSCL±P (p = 0.017; OR: 0.368; 95% CI: 0.152 - 0.893), with this particular single-nucleotide polymorphism present in 20% (20) of the controls and 7.9% (7) of the NSCL±P cases.

**Table 2:** Genotype and allele frequency of MSX1 variants and its association with NSCL±P

	NSCL±P patients n (%)	Controls n (%)	p-value*	OR (95% CI)
c.-36G>A			0.63	0.556
Genotype: GG	88 (98.9)	98 (98.0)		
Genotype: GA	1 (1.1)	2 (2.0)		
Allele: G	177 (99.4)	198 (99.0)		
Allele: A	1 (0.6)	2 (1.0)		
p. Ala30Ala			0.934	1.124
Genotype: CC	88 (98.9)	99 (99.0)		
Genotype: CA	1 (1.1)	1 (1.0)		
Allele: C	177 (99.4)	199 (99.5)		
Allele: A	1 (0.6)	1 (0.5)		
p. Ala34Gly			0.446	0.734
Genotype: CC	75 (88.8)	85 (85.0)		
Genotype: CG	10 (11.2)	15 (15.0)		
Allele: C	168 (94.4)	185 (92.5)		
Allele: G	10 (5.6)	15 (7.5)		
p.Gly110Gly			0.717	1.136
Genotype: CC	73 (82.0)	84 (84.0)		
Genotype: CT	16 (18.0)	16 (16.0)		
Allele: C	162 (91.0)	184 (92.0)		
Allele: T	16 (9.0)	16 (8.0)		
rs8670: C>T			0.017*	0.368
Genotype: CC	82 (92.1)	80 (80.0)		
Genotype: CT	7 (7.9)	20 (20.0)		
Allele: C	171 (96.1)	180 (90.0)		
Allele: T	7 (3.9)	20 (10.0)		
*Chi-square test, significant at p < 0.05; n = Number of patients/controls; OR = Odds ratio				

## Discussion

The majority of Malay individuals with clefts in this current study clinically presented with unilateral CLP. The number of subjects that had their left side affected was twice the number of those with their right side affected. The incidence of left unilateral CLP in some ethnic groups has been reported to be three times more frequent than on the right side of the face [2,16,19]. Although not conclusive, this phenomenon could be the result of better blood profusion from the blood vessel supplying the right side of the fetal head during early embryogenesis [20].

Despite several studies supporting the role of the MSX1 gene in the etiology of NSCL±P, no specific variants within this gene have been shown to be a common cause of clefts [2,7,10,11,13]. It appears that some variants are population-specific and may not be detectable in other ethnic groups. Interestingly, genetic risk factors for cleft are also influenced by environmental factors such as smoking, alcohol consumption, poor nutrition, and exposure to teratogens. In this case-control study, we investigated the contribution of variants within the MSX1 gene to oral clefts in the Malay population, the largest ethnic group in Malaysia. We report MSX1 gene variants in the Malay population, some of which have been previously reported in different Asian ethnicities (Table 3).

**Table 3:** Types of variants in the MSX1 gene involved in non-syndromic CL±P among different Asian ethnicities

Ethnicity	Variations in MSX1 gene	Reference
Thai	p.Ala34Gly; p.Pro147Gln; p.Gly267Cys; p.Pro278Ser	[11]
Vietnamese	c.-36G>A; p.Gly110Gly; p. Ala34Gly; p.Leu53Leu; p.Gly98Glu; p.Pro147Gln; rs8670: C>T; p.Gly267Cys	[10]
Filipino	p.Pro147Gln	[1]
Korean	p.Ala34Gly; p.Gly110Gly	[25]
Malay	p.Ala34Gly; p.Gly110Gly [#]; p.Pro147Gln; p.Gly267Ala; p.Met37Leu	[13]
	c.-36G>A; p.Ala30Ala; p.Ala34Gly; p.Gly110Gly; rs8670: C>T [*]	Current study
[*] Significant results in the current study but not in other studies; [#] Significant result in previous studies but not in the current study		

In contrast to a previous study on the Malay population, our study identified the rs8670: C>T variant near 3’ UTR of exon 2 in a total of 7 (7.9%) cleft patients and 20 (20%) healthy control subjects. Statistical analysis showed a significant genotype association between cleft cases and controls (p = 0.017; OR: 0.368; 95% CI: 0.152 - 0.893). Previous studies in other Asian populations have also shown a slightly higher heterozygous genotype (CT) for rs8670: C>T variant in control subjects, although the differences were statistically not significant [7,10], unlike the findings in the current study. This polymorphism, located 6 base pairs downstream of the stop codon in exon 2, may interfere with mRNA stability, regulation by MSX1 anti-sense RNA, or potentially alter the translational process [21]. However, the molecular mechanism underlying the pathogenesis or reduction in the incidence of cleft remains to be elucidated. A group of researchers reported six SNP groups comprising 12 SNPs (SG1: rs12532/rs8670/rs3775261/rs4464513; SG2: rs104893854 (P147Q); SG3: rs6446693/rs1907998; SG4: rs6832405/rs1042484; SG5: rs11726039/rs868257; and SG6: rs3821949) and reported that polymorphisms in groups 1 and 4 protected carriers from NSCL±P [22].

This study also observed c.-36G>A variant located in the 5’ UTR of the MSX1 gene in one patient with UCLP and two control subjects. However, this variant did not show any statistically significant difference. This c.-36G>A variant also did not show any significant difference between cases and controls in Caucasian populations [7,17]. It is therefore likely that this rare c.-36G>A variant does not play a significant role in contributing to the incidence of cleft.

Another MSX1 variant found in our study was the missense variant p.Ala34Gly, which results in an amino acid change from alanine to glycine. Finnerty et al. stated that this variant is situated within a series of alanine residues in the most divergent portion of the MSX1 gene sequence upstream of the most conserved domain [23]. Even so, the alanine repeats at the N-terminus of the MSX1 protein are believed to play an important role as a protein-protein interaction interface during the formation of transcriptional complexes [24,25]. Thus, the p.Ala34Gly mutation might disrupt such protein-protein interactions, consequently affecting downstream target gene transcription and ultimately the fusion of facial primordia. This p.Ala34Gly variant was previously reported in various other Asian populations [10,11,13,26], although it was generally considered benign. However, a study in the Nigerian population showed a significant association between p.Ala34Gly and NSCL±P (p-value < 0.05) [2].

A synonymous variant, p.Ala30Ala, was observed at a similar frequency in control and patient groups (1% and 1.1%, respectively) in this study. This variant was previously found in individuals of Filipino ethnicity but was notably absent in cleft patients and controls from South Americans and Caucasians [7]. Another synonymous variant, p.Gly110Gly, also located in the exon 1 of the MSX1 gene, was observed at a similar frequency in both groups. No significant association was observed in previous studies between the p.Gly110Gly variant and non-syndromic oral clefts [10,17,26]. However, a higher frequency of the p.Gly110Gly CT genotype was reported in a combined analysis of both CL±P and CPO cases, although no significant difference was found for CL±P cases alone [7].

Some limitations of this study include the relatively small sample size and the potential influence of environmental factors on the development of NSCL±P. Additionally, since NSCL±P is polygenic in nature, interaction between MSX1 and other genes may also play a role. Therefore, further studies with a larger sample size and consideration of environmental factors such as maternal smoking, alcohol use, and nutritional factors, along with other genes involved in NSCL±P, will provide more insight into the underlying mechanisms of the condition. 

Given the complex genetic contributions to NSCL±P, it will be important to identify further common variants as well as any de novo rare variants that contribute both positively and negatively to cleft incidence. Such detailed knowledge is critical for predicting inherent risk. To this end, genome-wide association studies (GWAS) and next-generation sequencing (NGS) can be utilized to shed some light on the identification of new susceptible genes as well as possible interaction among different genes and their variants that have been shown to play an important role during lip and palate morphogenesis [27,28]. Such understanding of both population-level and private variants is necessary to identify and address any interactions between specific environmental factors and each genetic variant [29,30]. It is only with such knowledge that effective interventional therapies may be possible for at-risk individuals by enabling personalized medicine approaches, where specific environmental factors and their interactions with genetic variants are considered to tailor prevention strategies and therapies for at-risk individuals.

## Conclusions

This study revealed a lower incidence of the rs8670: C>T polymorphism among NSCL±P patients compared to normal control subjects in this Malay population. However, it is unclear how this variant might exert its effect, although its location in the 3’UTR could potentially impact the stability of MSX1 mRNA. Future studies should focus on expanding the sample size and incorporating environmental factors, along with other genes involved in NSCL±P, to better understand the complex interactions contributing to this condition. 
